# β-Elemene inhibits the proliferation of primary human airway granulation fibroblasts by down-regulating canonical Wnt/β-catenin pathway

**DOI:** 10.1042/BSR20171386

**Published:** 2018-04-13

**Authors:** Cheng Xue, Ling-Ling Hong, Jun-Sheng Lin, Xiang-Yang Yao, Ding-Hui Wu, Xiao-Ping Lin, Jia-Min Zhang, Xiao-Bin Zhang, Yi-Ming Zeng

**Affiliations:** 1 Respiratory Medicine Center of Fujian Province, Department of Respiratory and Critical Care Medicine, The Second Affiliated Hospital of Fujian Medical University, Quanzhou, Fujian 362000, China; 2 Department of Respiratory Medicine, Zhangzhou Hospital of Traditional Chinese Medicine, Zhangzhou, Fujian 363401, China; 3 School of Biomedical Sciences, Huaqiao University, Quanzhou, Fujian 362000, China; 4 Department of Respiratory Medicine, The First Affiliated Hospital of Xiamen University, Xiamen, Fujian 361003, China; 5 Department of Respiratory Medicine, Zhongshan Hospital of Xiamen University, Xiamen, Fujian 361003, China

**Keywords:** β-Elemene, GSK-3β, Human airway granulation fibroblast, LiCl, α-SMA, Wnt/β-catenin

## Abstract

Benign airway stenosis is a clinical challenge because of recurrent granulation tissues. Our previous study proved that a Chinese drug, β-elemene, could effectively inhibit the growth of fibroblasts cultured from hyperplastic human airway granulation tissues, which could slow down the progression of this disease. The purpose of the present study is to find out the mechanism for this effect. We cultured fibroblasts from normal human airway tissues and human airway granulation tissues. These cells were cultured with 160 μg/ml normal saline (NS), different doses of β-elemene, or 10 ng/ml canonical Wnt/β-catenin pathway inhibitor (Dickkopf-1, DKK-1). The proliferation rate of cells and the expression of six molecules involved in canonical Wnt/β-catenin pathway, Wnt3a, glycogen synthase kinase-3β (GSK-3β), β-catenin, α-smooth muscle actin (α-SMA), transforming growth factor-β (TGF-β), and Collagen I (Col-I), were measured. At last, we used canonical Wnt/β-catenin pathway activator (LiCl) to further ascertain the mechanism of β-elemene. Canonical Wnt/β-catenin pathway is activated in human airway granulation fibroblasts. β-Elemene didn’t affect normal human airway fibroblasts; however, it had a dose–responsive inhibitive effect on the proliferation and expression of Wnt3a, non-active GSK-3β, β-catenin, α-SMA, TGF-β, and Col-I of human airway granulation fibroblasts. More importantly, it had the same effect on the expression and nuclear translocation of active β-catenin. All these effects were similar to 10 ng/ml DKK-1 and could be attenuated by 10 mM LiCl. Thus, β-elemene inhibits the proliferation of primary human airway granulation fibroblasts by down-regulating canonical Wnt/β-catenin pathway. This pathway is possibly a promising target to treat benign tracheobronchial stenosis.

## Introduction

Benign tracheobronchial stenosis is the narrow airway caused by nonmalignant factors, including tracheotomy, intubation, tuberculosis, systemic diseases, tracheobronchomalacia, and lung transplantation [1]. In China, the most common causes are tuberculosis and intubation [2]. With the development of interventional pulmonology, bronchoscope interventions (balloon dilatation, laser or electro resection, and stent placement) have replaced open surgery, being the main treatment for benign tracheobronchial stenosis [3]. However, the long-term treatment efficacy remains unsatisfactory. The narrow process recurs in many patients [4], which forces them to receive repeated bronchoscopic interventions. Why is the recurrence rate so high? The exuberant granulation is the main culprit [5]. Following a chemical or physical irritation, such as tuberculosis and intubation, the inflammatory cells are recruited and release numerous inflammatory factors, which can promote the proliferation of fibroblasts and vascular endothelial cells [6]. Then, the granulation tissues form [7] and make the airway narrow. This process repeats and makes the airway narrow repeatedly. Thus, how to effectively suppress the growth of airway granulation tissues, especially the burgeoning fibroblasts in them, has become a challenge to treat benign tracheobronchial stenosis.

Elemene is a Chinese traditional medicine isolated from the essential oils of Rhizoma Curcumae. β-Elemene is the main constituent of elemene and has been proved to be able to slow down the progression of a wide spectrum of malignant tumors [8]. However, many recent studies also showed that β-elemene could suppress fibrosis. β-Elemene was found to be effective in inhibiting the viability of fibroblasts cultured from the stiff joints and spines for patients with rheumatoid arthritis [9] and ankylosing spondylitis [10], which could slow down the progression of these two diseases. It was also effective in delaying the progression of atherosclerosis [11] and hepatic fibrosis [12]. Our previous study also found that β-elemene could effectively inhibit the growth of human airway granulation tissues and fibroblasts cultured from them, which could retard the progression of benign tracheobronchial stenosis [13]. However, the exact mechanism was still unknown.

Canonical Wnt/β-catenin pathway is an evolutionarily conserved signal pathway which plays an important role in promoting fibrosis in many organs, such as the lung, kidney, liver, heart, and skin [14]. We hypothesized that β-elemene inhibited the proliferation of primary human airway granulation fibroblasts via this pathway. Wnt3a is the ligand which can activate this pathway. Glycogen synthase kinase-3β (GSK-3β) is one molecule of destruction complex involved in this pathway. The destruction complex can phosphorylate a central molecule in this pathway, β-catenin, leading to β-catenin inactivation and degradation. When GSK-3β is phosphorylated, it loses activity leading to the destruction of the degradation complex. Thus, β-catenin cannot be phosphorylated and becomes active β-catenin. It will accumulate in the cytoplasm and translocate into the nucleus and then activate fibrosis-related genes’ transcription, such as α-smooth muscle actin (α-SMA), transforming growth factor-β (TGF-β), and Collagen I (Col-I). In the present study, we tested Wnt3a, GSK-3β, β-catenin, α-SMA, TGF-β, and Col-I to ascertain whether our hypothesis was correct.

## Materials and methods

### Reagents

High glucose Dulbecco’s modified Eagle’s medium (DMEM), 0.25% trypsin-EDTA, and FBS were purchased from HyClone (U.S.A.). MTT cell proliferation and cytotoxicity detection reagent kit were purchased from Keygen Biotech (Jiangsu, China). PrimeScript™ RT (RR037A) and SYBR^®^ premix Ex Taq™ (RR420A) reagent kits were purchased from TaKaRa (Japan). TGF-β and Col-I ELISA kits were purchased from Neobioscience (China) and Xlpcc (China) respectively. Primers of human GAPDH, Wnt3a, GSK-3β, β-catenin, α-SMA, TGF-β, and Col-I were purchased from Sangon Biotech (Shanghai, China). Primary monoclonal antibodies of human GAPDH (ab181602), Wnt3a (ab81614), and α-SMA (ab32575) were purchased from Abcam (U.S.A.) and GSK-3β (12456), phospho-GSK-3β (Ser9) (5558), β-catenin (8480), and non-phospho (active) β-catenin (Ser45) (19807) were purchased from Cell Signaling Technology (U.S.A.). Goat anti-rabbit and mouse horseradish peroxidase-conjugated secondary antibodies and goat anti-rabbit fluorescent secondary antibody were purchased from Beyotime (China). DAPI (1 mg/ml) was purchased from Sigma (U.S.A.). β-Elemene (98% in purity) purchased from Jingang Pharmaceutical Company (Dalian, China) was dissolved in high glucose DMEM to make a stock solution (5 mg/ml). Canonical Wnt/β-catenin pathway inhibitor (DKK-1) and activator (LiCl) were purchased from RD Systems (U.S.A.) and Sigma (U.S.A.) respectively. They were both dissolved in the same media to make a stock solution with the concentration of 100 ng/ml and 100 mg/ml respectively.

### Cell culture and treatments

The research plan followed guidelines of Helsinki Declaration and was endorsed by the Ethics Committee of the Second Affiliated Hospital of Fujian Medical University (approval No. 2015-007). After normal people and patients diagnosed with benign tracheobronchial stenosis signed informed consent forms, normal airway tissues and airway granulation tissues were obtained by small biopsy forceps under bronchoscopy examination. Fibroblasts were cultured from these two tissues using a reformative method called a combination of “tissue adherent” and “trypsinization” [15]. These cells were maintained in a medium containing 80% high glucose DMEM and 20% FBS and kept in an incubator with 5% CO_2_ and 37°C. They were passaged every 3 days and the cells for passage 5–7 were incubated in six-well plates, 96-well plates, or coverslips on six-well plates. After 48 h, these cells were cultured with 2% FBS supplemented with 160 μg/ml normal saline (NS), various doses of β-elemene (0, 40, 80, 120, and 160 μg/ml), or 10 ng/ml DKK-1. The fibroblasts cultured from granulation tissues were also cultured in the same media with various doses of LiCl (0, 5, 10, and 15 mM), or 160 μg/ml β-elemene plus 10 mM LiCl for 48 h.

### MTT assay

According to MTT cell proliferation and cytotoxicity detection reagent kit’s instructions, the cells cultured in 96-well plates were used for this experiment. Fifty microliters of 1× MTT was added into each well and the 96-well plate was maintained at 37°C for 4 h. Then the cell culture supernatant was removed and 150 μl of DMSO was added into each well. The optical density (OD) value for each well was measured by Microplate Reader (DeTia, China) at a wavelength of 490 nm. The experiment was repeated for at least three times.

### Quantitative real-time polymerase chain reaction (QRTPCR) analysis

Total RNA was extracted from fibroblasts using trizol under the manufacturer’s instructions. cDNA was synthesized from 500 ng of total RNA under PrimeScript™ RT reagent kit’s instructions. Two hundred nanograms of cDNA from each sample was used for quantitative real-time polymerase chain reaction (QRTPCR) under SYBR^®^ premix Ex Taq™ reagent kit’s instructions. The genes’ expression was detected by ABI PRISM 7500 sequence detection system (Applied Biosystems, U.S.A.). GAPDH was used as the internal control and fold changes of the genes’ expression were calculated by the method of 2^−ΔΔ*C*^_T_. The experiment was repeated for at least three times. Primers for QRTPCR were shown in Table 1.

**Table 1:** Primers used for QRTPCR

Gene (human)		Sequence (5′-3′)
GAPDH	Forward	CGGAGTCAACGGATTTGGTCGTAT
	Reverse	AGCCTTCTCCATGGTGGTGAAGAC
Wnt3a	Forward	AGATGGTGGTGGAGAAGCAC
	Reverse	TTGGGCTCGCAGAAGTTG
GSK-3β	Forward	AAGGTCCTGGGAACTCCAAC
	Reverse	GGGTCGGAAGACCTTAGTCC
β-Catenin	Forward	GCTGGTGACAGGGAAGACAT
	Reverse	CCATAGTGAAGGCGAACTGC
α-SMA	Forward	GCTGTTTTCCCATCCATTGT
	Reverse	TTTTGCTCTGTGCTTCGTCA
TGF-β	Forward	CTGGCGATACCTCAGCAAC
	Reverse	TAAGGCGAAAGCCCTCAAT
Col-I	Forward	AAGAGGCGAGAGAGGTTTCC
	Reverse	ACCAGCATCACCCTTAGCAC

### Western blot analysis

The cells were digested by 0.25% trypsin-EDTA and total proteins were extracted by cell lysis buffer supplemented with protease inhibitors. Equal amount of total proteins from each group was separated by SDS/PAGE and the proteins needed were transferred onto PVDF membranes. The membranes were incubated with 5% skimmed milk for 2 h, followed by primary antibodies of Wnt3a (1:2000), GSK-3β (1:1000), phospho-GSK-3β (Ser9) (1:1000), β-catenin (1:1000), and α-SMA (1:5000) overnight at 4°C. Subsequently, the membranes were washed and incubated with goat anti-rabbit (1:100000) or mouse (1:5000) horseradish peroxidase-conjugated secondary antibody for 1 h. Finally, the membranes were washed again and ECL detection reagent was added to make protein bands to be seen under ImageQuant LAS4000 chemiluminescence detection system (GE Healthcare, U.K.). The protein levels were quantified by ImageJ software and the gray values were normalized to GAPDH. The data were obtained from at least three independent experiments.

### Immunofluorescence staining analysis

The cells on coverslips after treatment were fixed by 4% paraformaldehyde for 20 min. Then, 0.1% Triton X-100 was added on the cells for 20 min to make cell membrane perforated. The cells were incubated with 5% BSA for 30 min, followed by primary antibodies of non-phospho (active) β-catenin (1:1600) overnight at 4°C. Subsequently, the cells were washed for three times and incubated with goat anti-rabbit fluorescent secondary antibody (1:400) in dark for 1 h. The cells were washed three times again and incubated with DAPI (0.1 μg/ml) in dark for 10 min to make nuclear dyed. Finally, the cells were washed for four times and mounting medium containing anti-fluorescence quenching agent was added after the coverslips were dried. The cells’ fluorescence was observed under fluorescence microscope (Olympus, Japan).

### ELISA assay

The cell culture supernatant was used for ELISA assay. The concentrations of TGF-β and Col-I were measured according to ELISA kits’ protocols. The experiment was repeated for at least three times.

### Statistical analysis

The data were analyzed by one-way variance analysis followed by Tukey’s *post hoc* test for three or more groups’ comparison or Student’s *t* test for two groups’ comparison using Statistical Product and Service Solutions (SPSS) software 21.0 (Chicago, U.S.A.) and were expressed as the mean and standard deviation (SD). *P*<0.05 indicated that the difference between two groups was statistically significant and *P*<0.01 indicated that the difference was highly significant.

## Results

### Canonical Wnt/β-catenin pathway is activated in primary human airway granulation fibroblasts

**Figure 1:**
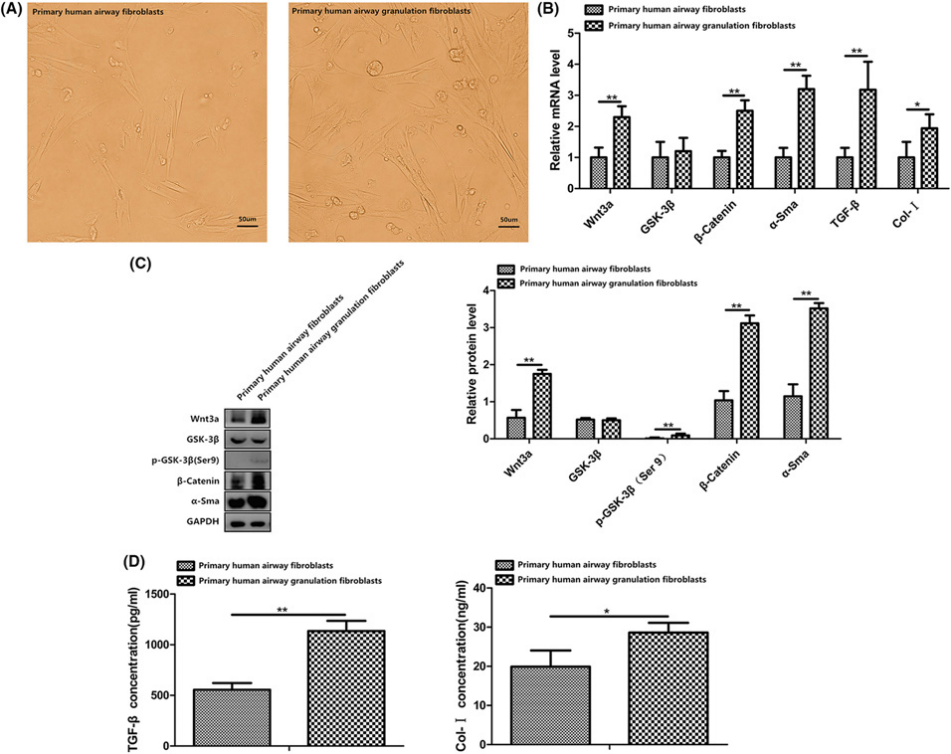
The morphology and expression of Wnt3a, GSK-3β, β-catenin, α-SMA, TGF-β, and Col-I of primary human airway fibroblasts and primary human airway granulation fibroblasts (**A**) Primary human airway fibroblasts and primary human airway granulation fibroblasts were cultured in media containing 80% high glucose DMEM and 20% FBS. The morphology of cells was shown in photomicrographs (200× magnification). (**B**) The mRNA levels of Wnt3a, GSK-3β, β-catenin, α-SMA, TGF-β, and Col-I were assessed by QRTPCR. (**C**) The protein levels of Wnt3a, GSK-3β, phospho-GSK-3β (Ser9), β-catenin, and α-SMA were assessed by Western blot. (**D**) The protein levels of TGF-β and Col-I were assessed by ELISA. GAPDH was used as the internal control and the data of three independent experiments were expressed as the mean ± SD. (*P < 0.05, **P < 0.01).

**Figure 2:**
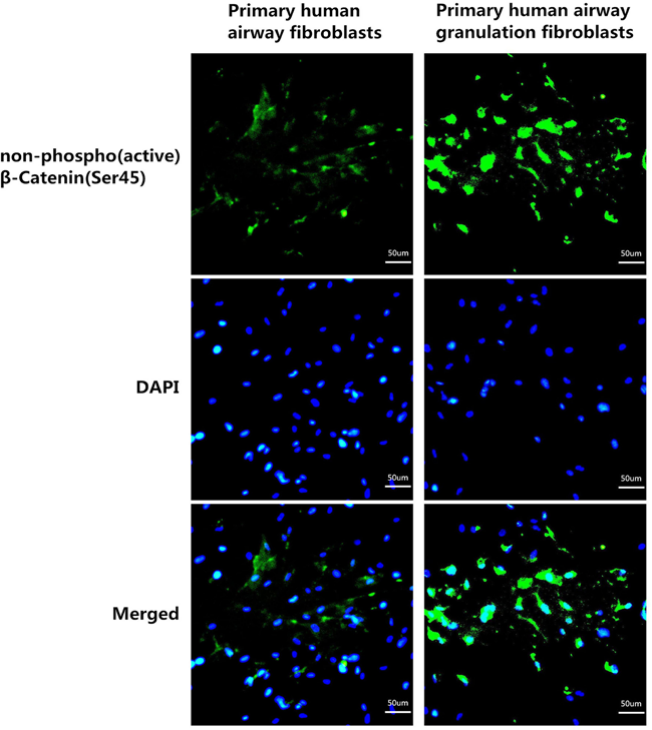
The expression and nuclear translocation of non-phospho (active) *β*-catenin (Ser45) of primary human airway fibroblasts and primary human airway granulation fibroblasts Primary human airway fibroblasts and primary human airway granulation fibroblasts were cultured in media containing 80% high glucose DMEM and 20% FBS. The protein level and nuclear translocation of non-phospho (active) *β*-catenin (Ser45) were assessed by immunofluorescence staining (200× magnification).

Fibroblasts cultured from normal human airway tissues and human airway granulation tissues were almost the same. However, after a careful observation, we could see a bit different. Primary human airway granulation fibroblasts are bigger and longer and have more branches compared with primary human airway fibroblasts (Figure 1A). We first examined whether this pathway is activated in primary human airway granulation fibroblasts. mRNA and proteins were extracted from primary human airway fibroblasts and primary human airway granulation fibroblasts. The above six molecules were assessed by QRTPCR, Western blot, immunofluorescence staining, and ELISA. The mRNA and protein levels of Wnt3a, β-catenin, α-SMA, TGF-β, and Col-I were up-regulated in primary human airway granulation fibroblasts. There were no significant difference of the mRNA and protein levels of GSK-3β between these two cells. The phosphorylated form of GSK-3β and the unphosphorylated form of β-catenin were up-regulated and more unphosphorylated β-catenin translocated into the nucleus (Figures 1B–D and 2). These results indicated that canonical Wnt/β-catenin pathway is activated in primary human airway granulation fibroblasts.

### β-Elemene inhibited the proliferation of primary human airway granulation fibroblasts

**Figure 3:**
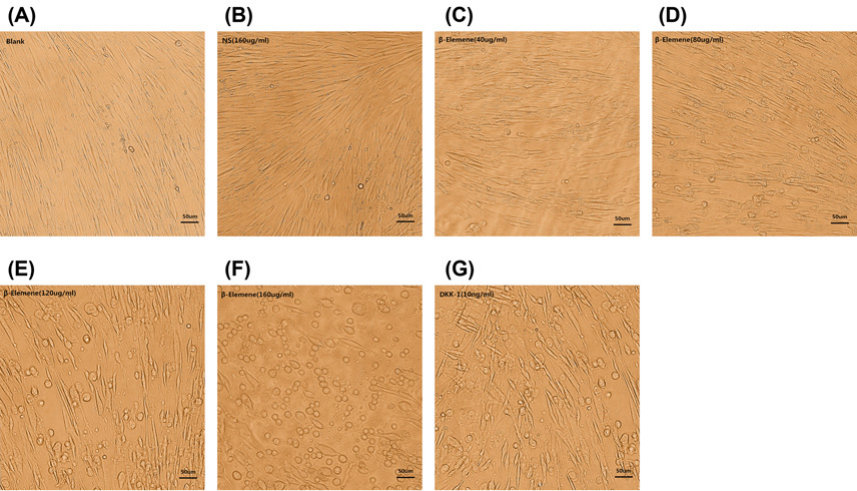
β-Elemene had a dose–responsive inhibitive effect on the proliferation of primary human airway granulation fibroblasts (**A, B, C, D, E, F**, and **G**) Primary human airway granulation fibroblasts were cultured in media containing 2% FBS supplemented with 160 μg/ml NS, various doses of β-elemene (0, 40, 80, 120, and 160 μg/ml), or 10 ng/ml DKK-1 for 48 h. The morphology of cells was shown in photomicrographs (200× magnification).

**Figure 4:**
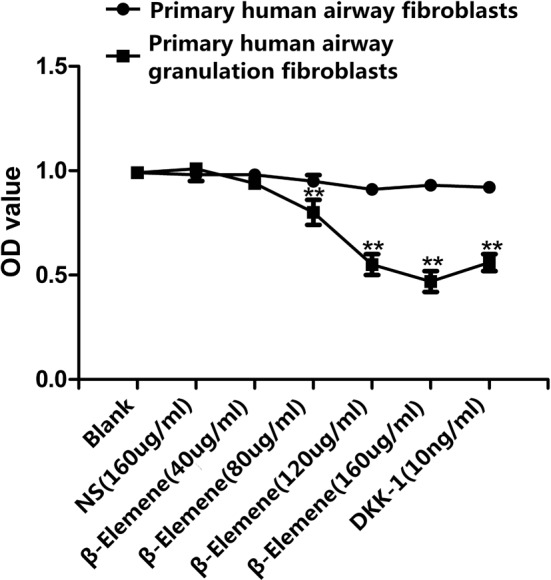
The effect of β-elemene on the proliferation of primary human airway fibroblasts and primary human airway granulation fibroblasts Primary human airway fibroblasts and primary human airway granulation fibroblasts were cultured in media containing 2% FBS supplemented with 160 μg/ml NS, various doses of β-elemene (0, 40, 80, 120, and 160 μg/ml), or 10 ng/ml DKK-1 for 48 h. The proliferation rate of cells was assessed by MTT assay. The data of three independent experiments were expressed as the mean ± SD (**P<0.01 compared with the control group).

After treatment with different concentrations of β-elemene for 48 h, we observed distinct morphological changes of primary human airway granulation fibroblasts under the microscope. The cells not treated with β-elemene showed a typical spindle-shaped morphology and arranged orderly and closely (Figure 3A and B). At the concentration of 40 μg/ml, the cells showed a slight irregular arrangement. Some of them shrank and became round. The intercellular space enlarged and more cytoplasmic particles were observed (Figure 3C). At the concentration of 80 μg/ml, more cells shrank and became round with larger intercellular spaces (Figure 3D). At the concentration of 120 μg/ml, most cells shrank and became round and the intercellular space became enormous (Figure 3E). At the concentration of 160 μg/ml, almost all cells became round and few typical spindle-shaped cells remained. Numerous cells died and floated up (Figure 3F). MTT assay showed the same results. After being treated with 40, 80, 120, and 160 μg/ml β-elemene for 48 h, the proliferation rate of cells decreased by 6.93%, 20.79%, 45.54%, and 53.47% respectively. However, β-elemene showed no significant inhibitive effect on the proliferation of primary human airway fibroblasts (Figure 4). All these effects were similar to the pathway inhibitor, DKK-1 (Figure 3G). These results indicated that β-elemene had a dose–responsive inhibitive effect on the proliferation of human airway granulation fibroblasts and didn’t affect normal human airway fibroblasts.

### β-Elemene inhibited canonical Wnt/β-catenin pathway of primary human airway granulation fibroblasts

To determine whether this pathway is underlying the inhibitive effect of β-elemene on primary human airway granulation fibroblasts, mRNA and proteins were extracted from primary human airway granulation fibroblasts treated with 160 μg/ml NS, different concentrations of β-elemene, or 10 ng/ml DKK-1. The above six molecules were assessed by QRTPCR, Western blot, immunofluorescence staining, and ELISA.

**Figure 5:**
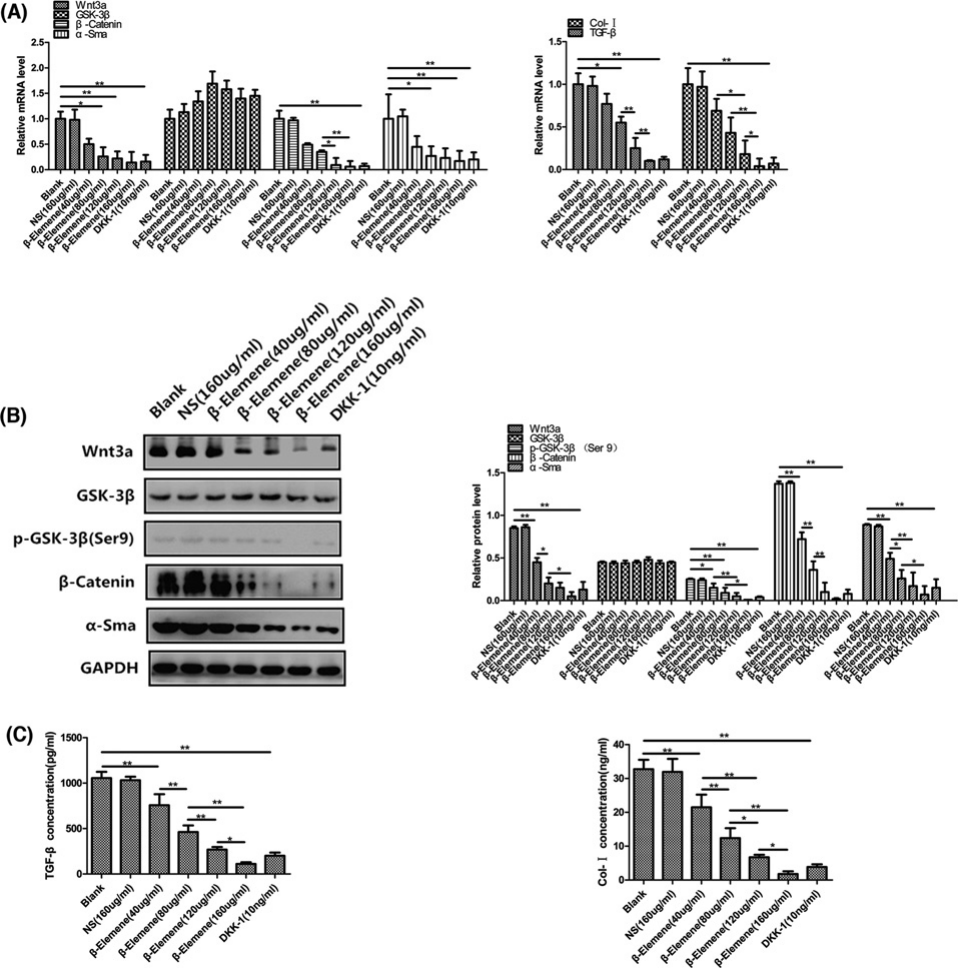
The effect of β-elemene on the expression of Wnt3a, GSK-3β, β-catenin, α-SMA, TGF-β, and Col-I of primary human airway granulation fibroblasts (**A**) Primary human airway granulation fibroblasts were cultured in media containing 2% FBS supplemented with 160 μg/ml NS, various doses of β-elemene (0, 40, 80, 120, and 160 μg/ml), or 10 ng/ml DKK-1 for 48 h. The mRNA levels of Wnt3a, GSK-3β, β-catenin, α-SMA, TGF-β, and Col-I were assessed by QRTPCR. (**B**) The protein levels of Wnt3a, GSK-3β, phospho-GSK-3β (Ser9), β-catenin, and α-SMA were assessed by Western blot. (**C**) The protein levels of TGF-β and Col-I were assessed by ELISA. GAPDH was used as the internal control and the data of three independent experiments were expressed as the mean ± SD (*P<0.05, **P<0.01).

β-Elemene had a significant dose–responsive inhibitive effect on the mRNA and protein levels of Wnt3a (Figure 5A and B). After being treated with 40, 80, 120, and 160 μg/ml β-elemene for 48 h, Wnt3a mRNA levels decreased by 50.00%, 74.00%, 78.00%, and 86.00% respectively (Figure 5A) and Wnt3a protein levels decreased by 47.06%, 76.47%, 82.35%, and 94.12% respectively (Figure 5B).

The mRNA and protein levels of GSK-3β of primary human airway granulation fibroblasts were not affected by β-elemene. However, β-elemene had a significant dose–responsive inhibitive effect on the phosphorylated protein level of GSK-3β (Figure 5A and B). As shown in Figure 5B, after being treated with 40, 80, 120, and 160 μg/ml β-elemene for 48 h, the phosphorylated protein levels of GSK-3β decreased by 40.00%, 64.00%, 80.00%, and 96.00% respectively.

**Figure 6:**
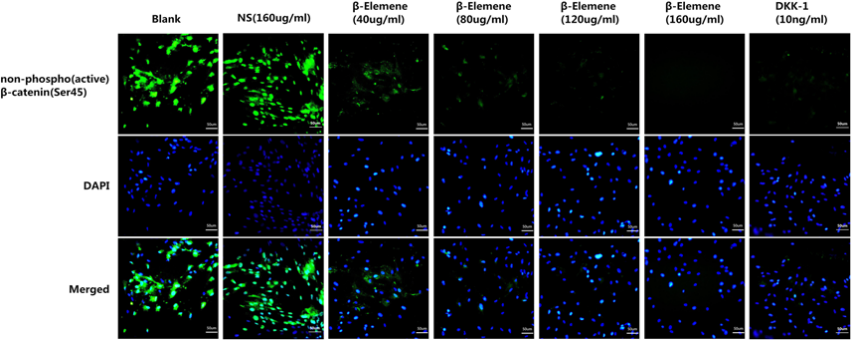
The effect of β-elemene on the expression and nuclear translocation of non-phospho (active) β-catenin (Ser45) of primary human airway granulation fibroblasts Primary human airway granulation fibroblasts were cultured in media containing 2% FBS supplemented with 160 μg/ml NS, various doses of β-elemene (0, 40, 80, 120, and 160 μg/ml), or 10 ng/ml DKK-1 for 48 h. The protein level and nuclear translocation of non-phospho (active) *β*-catenin (Ser45) were assessed by immunofluorescence staining (200× magnification).

β-Elemene had a significant dose–responsive inhibitive effect on the mRNA and protein levels of β-catenin (Figure 5A and B). After being treated with 40, 80, 120, and 160 μg/ml β-elemene for 48 h, β-catenin mRNA levels decreased by 51.00%, 65.00%, 91.00%, and 94.00% respectively (Figure 5A) and β-catenin protein levels decreased by 47.45%, 73.72%, 92.70%, and 98.54% respectively (Figure 5B). And the most important, β-elemene had a significant dose–responsive inhibitive effect on the protein level and nuclear translocation of unphosphorylated β-catenin (Figure 6).

β-Elemene had a significant dose–responsive inhibitive effect on the mRNA and protein levels of α-SMA (Figure 5A and B). After being treated with 40, 80, 120, and 160 μg/ml β-elemene for 48 h, α-SMA mRNA levels decreased by 55.00%, 73.00%, 77.00%, and 83.00% respectively (Figure 5A) and α-SMA protein levels decreased by 44.94%, 70.79%, 80.90%, and 92.13% respectively (Figure 5B).

β-Elemene had a significant dose–responsive inhibitive effect on the mRNA and protein levels of TGF-β (Figure 5A and C). After being treated with 40, 80, 120, and 160 μg/ml β-elemene for 48 h, TGF-β mRNA levels decreased by 23.00%, 45.00%, 75.00%, and 90.00% respectively (Figure 5A) and TGF-β protein levels decreased by 28.21%, 56.20%, 74.60%, and 89.50% respectively (Figure 5C).

β-Elemene had a significant dose–responsive inhibitive effect on the mRNA and protein levels of Col-I (Figure 5A and C). After being treated with 40, 80, 120, and 160 μg/ml β-elemene for 48 h, Col-I mRNA levels decreased by 31.00%, 57.00%, 82.00%, and 96.00% respectively (Figure 5A) and Col-I protein levels decreased by 34.41%, 62.20%, 79.51%, and 94.60% respectively (Figure 5C).

It was found that β-elemene up-regulated the activity of GSK-3β and down-regulated the activity of Wnt3a, β-catenin, α-SMA, TGF-β, and Col-I, all in a dose-dependent manner. All these effects were similar to the pathway inhibitor, 10 ng/ml DKK-1 (Figures 5 and 6). These results indicated that down-regulating the activity of canonical Wnt/β-catenin pathway is possibly the key mechanism by which β-elemene inhibits the proliferation of primary human airway granulation fibroblasts.

### LiCl attenuated the inhibitive effect of β-elemene on the proliferation and canonical Wnt/β-catenin pathway of primary human airway granulation fibroblasts

**Figure 7:**
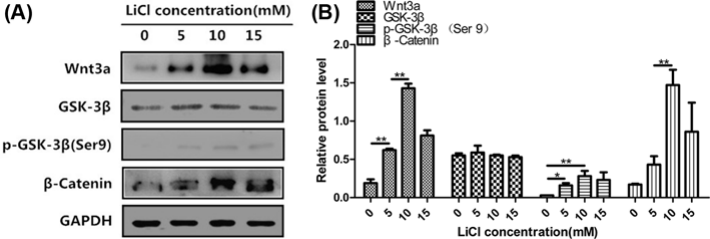
The effect of LiCl on the expression of Wnt3a, GSK-3β, and β-catenin of primary human airway granulation fibroblasts (**A** and **B**) Primary human airway granulation fibroblasts were cultured in media containing 2% FBS and 0, 5, 10, and 15 mM LiCl for 48 h. The protein levels of Wnt3a, GSK-3β, phospho-GSK-3β(Ser9), and β-catenin were assessed by Western blot. GAPDH was used as the internal control and the data of three independent experiments were expressed as the mean ± SD (*P<0.05, **P<0.01).

**Figure 8:**
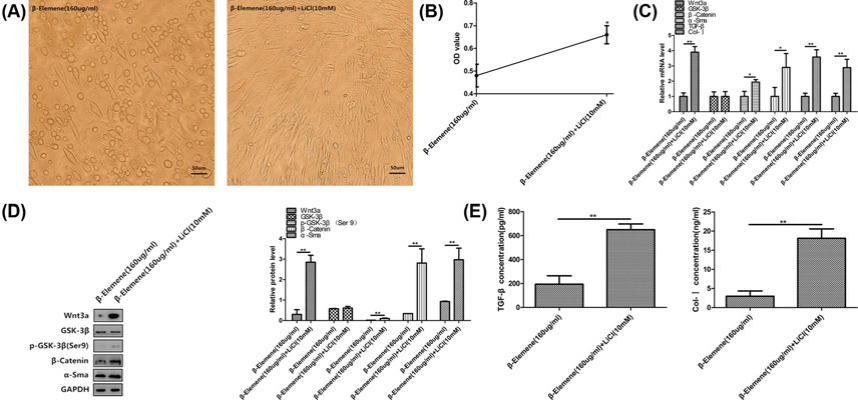
LiCl attenuated the inhibitive effect of β-elemene on the proliferation and canonical Wnt/β-catenin pathway of primary human airway granulation fibroblasts (**A**) Primary human airway granulation fibroblasts were cultured in media containing 2% FBS and 160 μg/ml β-elemene in the presence or absence of 10 mM LiCl for 48 h. The morphology of cells was shown in photomicrographs (200× magnification). (**B**) The proliferation rate of cells was assessed by MTT assay. (**C**) The mRNA levels of Wnt3a, GSK-3β, β-catenin, α-SMA, TGF-β, and Col-I were assessed by QRTPCR. (**D**) The protein levels of Wnt3a, GSK-3β, phospho-GSK-3β (Ser9), β-catenin, and α-SMA were assessed by Western blot. (**E**) The protein levels of TGF-β and Col-I were assessed by ELISA. GAPDH was used as the internal control and the data of three independent experiments were expressed as the mean ± SD (*P<0.05, **P<0.01).

**Figure 9:**
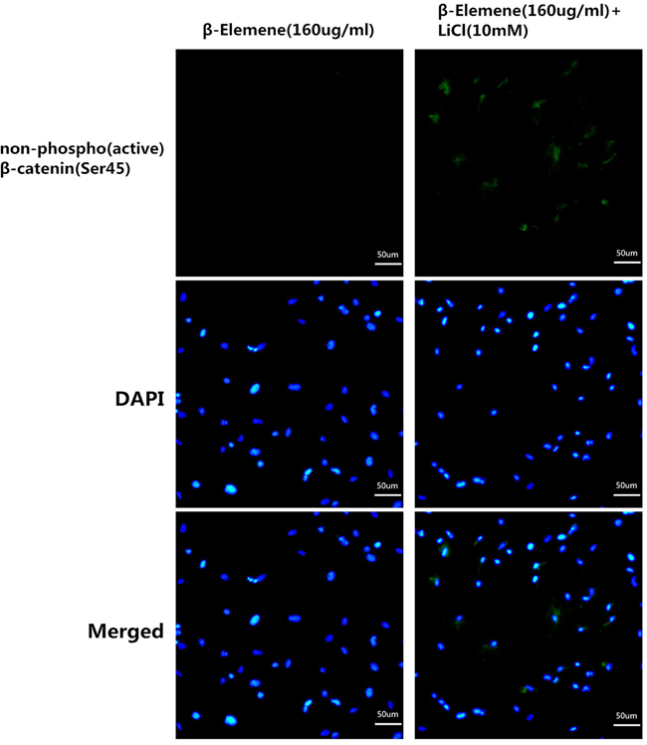
LiCl attenuated the inhibitive effect of β-elemene on the expression and nuclear translocation of non-phospho (active) β-catenin (Ser45) of primary human airway granulation fibroblasts Primary human airway granulation fibroblasts were cultured in media containing 2% FBS and 160 μg/ml β-elemene in the presence or absence of 10 mM LiCl for 48 h. The protein level and nuclear translocation of non-phospho (active) β-catenin (Ser45) were assessed by immunofluorescence staining (200× magnification).

At last, we used the pathway activator, LiCl, to further ascertain whether inhibiting canonical Wnt/β-catenin pathway is the key mechanism by which β-elemene inhibits the proliferation of primary human airway granulation fibroblasts. We first cultured primary human airway granulation fibroblasts with different concentrations of LiCl and found out 10 mM LiCl has the largest promotive effect on this pathway (Figure 7). Then, we cultured the cells with 160 μg/ml β-elemene in the presence or absence of 10 mM LiCl for 48 h. Compared with 160 μg/ml β-elemene treatment group, the cells treated with 160 μg/ml β-elemene plus 10 mM LiCl showed a more typical spindle-shaped morphology, with smaller intercellular spaces (Figure 8A). The proliferation rate of cells increased (Figure 8B). Similarly, the protein level of phosphorylated GSK-3β and mRNA and protein levels of Wnt3a, β-catenin, α-SMA, TGF-β, and Col-I increased (Figure 8C–E). And more importantly, the protein level and nuclear translocation of unphosphorylated β-catenin increased (Figure 9). These results proved that LiCl attenuated the inhibitive effect of β-elemene on the proliferation and canonical Wnt/β-catenin pathway of primary human airway granulation fibroblasts, which further demonstrated that down-regulating the activity of canonical Wnt/β-catenin pathway is the key mechanism by which β-elemene inhibits these cells.

## Discussion

Benign airway stenosis is easily to recur because of exuberant granulation tissues. One way to solve this problem is to inhibit the growth of airway granulation fibroblasts. β-Elemene is a widely used anticancer Chinese drug. Recently, many studies proved that it can also inhibit fibrosis. In the present study, we cultured normal human airway fibroblasts and human airway granulation fibroblasts with different concentrations of β-elemene and found out at the concentration equal or below 160 μg/ml, β-elemene had a dose–responsive inhibitive effect on the proliferation of human airway granulation fibroblasts. When the concentration reached to 160 μg/ml, about half of the cells were dead. And in this concentration range, β-elemene almost had no inhibitive effect on the proliferation of normal human airway fibroblasts. This indicated that β-elemene is a safe drug, and has specific inhibitive effect on human airway granulation fibroblasts. This drug can be a promising drug to treat benign airway stenosis.

Canonical Wnt/β-catenin pathway plays a vital role in regulating tissues’ regeneration [16]. This pathway is inactive under normal conditions. In hyperplastic diseases, such as tumorigenesis and fibrosis, this pathway is activated [17]. Our study proved that this pathway is activated in primary human airway granulation fibroblasts, which was in accordance with above studies. The mechanism of β-elemene to inhibit cancer or other diseases has been proven to be involved in many pathways, such as BMP/SMADs pathway, RhoA/ROCK pathway, and canonical Wnt/β-catenin pathway [18]. As canonical Wnt/β-catenin pathway is also related to fibrosis, we hypothesized that it is also involved in the inhibitive effect of β-elemene on primary human airway granulation fibroblasts.

β-Catenin is an important molecule related to fibrosis. Sustained activation of β-catenin would promote the proliferation of fibroblasts [19] and lead to hyperplastic cutaneous wounds in transgenic mice [20], while β-catenin knockout would produce adverse effects [21].

α-SMA is another molecule closely related to fibrosis. It is the main molecular marker of myofibroblasts [22]. Myofibroblasts are a cohort of fibroblasts characterized by excessive extracellular matrix synthesis and strong contractility [23]. They are important in normal healing processes and sustained activation of myofibroblasts may lead to fibrosis [24]. Myofibroblasts exist in nearly all kinds of fibrotic tissues and have been reported to exist in the narrow trachea [25]. A reduction in α-SMA expression will make myofibroblasts apoptosis and lose contractility [26], thus will inhibit fibrosis.

TGF-β and Col-I are two important molecules secreted by fibroblasts. TGF-β is one of the most potent cytokines to promote fibrosis and can promote fibroblasts to turn into myofibroblasts [27]. Col-I is one component of fibrous tissues’ extracellular matrix. Active fibroblasts can secrete a large amount of Col-I, which can promote fibrosis [28].

When canonical Wnt/β-catenin pathway is inactive, β-catenin will be phosphorylated by a destruction complex which includes adenomatous polyposis coli protein (APC), casein kinase 1 (CK1), AXIN, and GSK-3β. Then, β-catenin will be phosphorylated at serine residue 45 and then be ubiquitylated and degraded by proteasomes [29]. When canonical Wnt/β-catenin pathway is activated, Wnt ligands, such as Wnt3a, bind to the receptor. Then, GSK-3β will be phosphorylated at serine residue 9, makes its activity decreased by 30–70% [30]. This will cause the destruction of the degradation complex. As a result, β-catenin cannot be phosphorylated and degraded [31]. Unphosphorylated β-catenin is the active form of β-catenin. It will accumulate in the cytoplasm and translocate into the nucleus [32] and then activate target genes’ transcription [33]. Many of these genes are oncogenes and fibrosis-related genes, such as α-SMA, TGF-β, and Col-I. The mechanism for β-elemene to inhibit this pathway may first happen to inhibit the expression of Wnt3a. This causes the activity of GSK-3β decrease and the stabilization of the degradation complex, leading to the promotion of β-catenin phosphorylation and the inhibition of β-catenin nuclear translocation. And finally, the transcription of fibrosis-related gene, α-SMA, TGF-β, and Col-I, was inhibited.

Our study proved that β-elemene inhibited canonical Wnt/β-catenin pathway of primary human airway granulation fibroblasts, and this effect could be attenuated by the pathway activator, LiCl. This indicated that down-regulating the activity of canonical Wnt/β-catenin pathway is possibly the key mechanism by which β-elemene inhibits the proliferation of human airway granulation fibroblasts. This pathway may become a promising target to treat benign tracheobronchial stenosis.

## Conclusion

Canonical Wnt/β-catenin pathway is activated in primary human airway granulation fibroblasts. β-Elemene had a dose–responsive inhibitive effect on the proliferation of human airway granulation fibroblasts and didn’t affect normal human airway fibroblasts. It also had a dose–responsive inhibitive effect on the activity of canonical Wnt/β-catenin pathway of human airway granulation fibroblasts. All these effects were similar to 10 ng/ml DKK-1 and could be attenuated by 10 mM LiCl. These results indicated that down-regulating canonical Wnt/β-catenin pathway is possibly the key mechanism by which β-elemene inhibits the proliferation of primary human airway granulation fibroblasts.

## Abbreviations

α-SMA: α-smooth muscle actin
APC: adenomatous polyposis coli protein
AXIN: axin protein
BSA: bovine serum albumin
CK1: casein kinase 1
Col-I: Collagen I
DAPI: 4′,6-diamidino-2-phenylindole
DKK-1: Dickkopf-1
DMEM: Dulbecco’s modified Eagle’s medium
DMSO: dimethyl sulfoxide
ELISA: enzyme-linked immunosorbent assay
FBS: fetal bovine serum
GSK-3β: glycogen synthase kinase-3β
LiCl: lithium chloride
MTT: 3-(4,5-dimethyl-2-thiazolyl)-2,5-diphenyl-2-H-tetrazolium bromide
NS: normal saline
OD: optical density
PVDF: polyvinylidene fluoride
QRTPCR: quantitative real-time polymerase chain reaction
SD: standard deviation
SDS/PAGE: sodium dodecyl sulfate-polyacrylamide gel electrophoresis
SPSS: Statistical Product and Service Solutions
TGF-β: transforming growth factor-β
